# Site-selective solid phase synthesis of carbonylated peptides

**DOI:** 10.1007/s00726-015-1967-4

**Published:** 2015-03-27

**Authors:** Mateusz Waliczek, Monika Kijewska, Piotr Stefanowicz, Zbigniew Szewczuk

**Affiliations:** Faculty of Chemistry, University of Wrocław, F. Joliot-Curie 14, 50-383 Wrocław, Poland

**Keywords:** Oxidative stress, Oxidation, Carbonylation, Solid phase peptide synthesis, Carbonylated peptides, Posttranslational modification

## Abstract

The aim of our research was to design an efficient method for the solid phase synthesis of carbonylated peptides. For this purpose, we designed and synthesized a fully protected derivative Fmoc-amino(2,5,5-trimetyhyl-1,3-dioxolan-2-yl)acetic acid (Fmoc-Atda-OH) of a novel unnatural amino acid (Thr(O)-2-amino-3-oxo-butanoic acid). To obtain the mentioned derivative, two synthetic strategies were investigated using different reagents for carbonyl protection, ethane-1,2-diol and 2,2-dimethyl-propane-1,3-diol. The racemization of oxidized threonine was also analyzed and discussed. We successfully carried out the solid phase synthesis of peptides containing a Thr(O) moiety using Fmoc-Atda-OH according to the standard Fmoc strategy. The application of the designed building block allows the synthesis of peptides containing D,L**-**Thr(O) residue, which may be used as models of oxidatively modified peptides which occur in biological systems and are related to many diseases.

**Electronic supplementary material:**

The online version of this article (doi:10.1007/s00726-015-1967-4) contains supplementary material, which is available to authorized users.

## Introduction

The Reactive Oxygen Species (ROS) formation is the consequence of a redox regulation in cells (Madian and Regnier 2010a). The impaired balance between production and removing ROS is an undesirable phenomenon that can lead not only to oxidation of proteins but also other biomolecules, like DNA, fatty acids, and carbohydrates. The proteins, however, are the most susceptible to ROS impact. Many changes in amino acid residues resulting from oxidation reactions have been characterized, such as carbonylation or disulfide formation (Madian and Regnier 2010b). The selected examples are presented in Table 1 (Madian and Regnier 2010a). These post-translational modifications can be divided into three groups distinguishable by mass spectrometry. One involves oxidative cleavage in either the protein backbone or amino acid side chains, with Pro, Arg, Lys, Thr, Glu or Asp residues most likely to undergo oxidative cleavage. The Cys and Met are most susceptible to oxidative modification. Other moieties, especially Lys, Arg, Pro and Thr incur formation of carbonyl groups (aldehydes and ketones) in the side chains. This modification is based on the addition of lipid oxidation products such as 4-hydroxy-2-nonenal to proteins. Finally, carbonyl groups in proteins can be generated by advanced glycation.

**Table 1:** List of the different types of oxidative modifications (Madian and Regnier 2010a)

Amino acids	Oxidative modification
T	2-Amino-3-oxo-butanoic acid
Y, D, K, F, P	Hydroxylation
K	Aminoadipic semialdehyde, Amadori products, 3-Deoxyglucosone adducts, glyoxal adducts, methylglyoxal adducts, hydroxynonenal, malonodialdehyde, *N*-homocysteinylation
R	Glutamic semialdehyde
C	Sulfonic acid, sulfinic acid, sulfenic acid, S-homocysteinylation
M	Sulfoxide, sulfon
P	Glutamic semialdehyde, pyroglutamic

Some of these oxidations are reversible and can play a role in metabolism, the other—irreversible—often lead to protein inactivation. Protein carbonylation is considered to be the most common protein oxidation reaction, therefore, it has become an indicator of the oxidative stress (Moller and Rogowska-Wrzesińska 2011; Roe et al. 2010). It has been proven that metal-catalyzed oxidation (MCO) is the main mechanism of protein carbonylation. The other pathways leading to formation of carbonyl moieties involve either oxidative cleavage of proteins by α-amidation pathway or oxidation of glutamyl side chains (Stadtman and Levine 2003; Requena et al. 2001). An addition of 4-hydroxynoneal, the main product of lipid peroxidation, to nucleophilic groups in the side chains of amino acids is another mechanism leading to formation of the carbonylated proteins (Dalle-Donne et al. 2006). This post-translational modification (PTM) is linked to numerous diseases, including diabetes (Telci et al. 2000), cancer or Parkinson and Alzheimer diseases (Smith et al. 1991; Uttara et al. 2009).

Characterization of carbonylated proteins has been a challenge for contemporary proteomics due to their low abundance in the biological material as well as a diversity of possible modifications resulting from various pathways leading to the carbonyl compound formation. Because of the requirements imposed by proteomics, many useful methods allowing selecting and recognizing the carbonylated proteins have emerged. Most of these techniques are based on formation of Schiff bases (Dalle-Donne et al. 2005; Grimsrud et al. 2008) ranging from the classic dinitrophenylhydrazine derivatization (DNPH) (Palmese et al. 2011), through biotin hydrazide (Mirzaei et al. 2008) method to Girard’s P reagent (Mirzaei and Regnier 2006) and Oxidation-Dependent Element Coded Affinity Tags (O-ECAT) (Lee et al. 2006). The widely used enrichment approach consists reaction of the carbonyl group with biotin hydrazide and a further selection, by means of immobilized avidin or streptavidin on an affinity column (Mirzaei et al. 2008). The additional advantage of Girard’s P reagent, which is also based on hydrazone formation, is an increase in ionization efficiency resulting from the presence of a permanent positive charge in the quaternary ammonium moiety (Mirzaei and Regnier 2006). The high-resolution mass spectrometry combined with stable isotope labeling involving specific incorporation of ^18^O into the carbonyl moieties is also used for studying carbonylated proteins. The resulting isotopic signature observed in a mass spectrum allows detection of carbonylated peptides (Roe et al. 2010). Similarly, it was proven that treating the sample containing glycated peptides with H_2_^18^O under microwave activation, results in the isotopic exchange of anomeric oxygen which allows detection of the Amadori products by MS techniques (Kijewska et al. 2011). DNPH derivatized carbonylated peptides were also analyzed by ESI–MS/MS in the negative ion mode allowing a differentiation of various types of carbonyl compounds through a specific fragmentation (Bollineni et al. 2011a).

In view of the reasons given above, there is a need for new methods of carbonylated peptide synthesis, since they are useful as standards for quantitative analysis and help in development of enrichment methods. Actually, the available methods allow only for synthesis of model unnatural carbonylated peptides. Marceau (2005) and co-workers (Buré et al. 2012; Bollineni et al. 2011b) reported synthesis of the C-terminal alpha-oxo aldehyde peptides and pyruvic acid-containing peptides using solid phase synthesis. Another approach, based on periodate oxidation of 2-aminoalcohol (for example serine or threonine), results in formation of the aldehyde group at the N-terminus of a peptide chain.

The objective of our current work is a synthesis of peptides containing oxidized threonine using a fully protected building block. This approach allows preparation of analytically pure product containing Thr(O) residue. According to literature reports (Madian and Regnier 2010b), such peptides were detected in products of enzymatic hydrolysis of oxidized proteins. This synthetic approach is similar to that applied in our previous paper, focused on the reaction of a lysine moiety with reducing sugars (Stefanowicz et al. 2010). In the current project, we extended our research to the physiologically relevant oxidative modifications resulting in carbonylation of proteins. It may be expected that these model compounds will be useful for development and testing affinity-based procedures of preconcentration of carbonylated peptides. In addition, isotopically labeled analytically pure peptides containing carbonyl groups may be used for the quantitative determination of this modification in enzymatic digests.

## Experimental

### Reagents

Z-Thr-OBzl (>98 %) was purchased from Alfa Aesar (Karlsruhe, Germany). The oxidizing agents: pyridinium chlorochromate (PCC) was obtained from Sigma-Aldrich (St. Louis, MO, USA) and sulfur trioxide-pyridine complex was obtained from Fluka (Steinheim, Germany). The solvents used for Fmoc-Amda-OH synthesis were: dichloromethane (Chempur, Piekary Śląskie, Poland), dimethyl sulfoxide (Scientific Limited Park, Northapton, U.K), methanol (POCh, Poland), ethanol (Eurochem BGD), acetone (POCh, Poland), benzene (Eurochem BGD, Tarnów, Poland) and diethyl ether (Chempur, Poland). Ethylene glycol, 2,2-dimethyl-propane-1,3-diol and *p*-toluenesulfonic acid as a catalyst were obtained from Sigma-Aldrich (St. Louis, MO, USA). The protecting group 9-fluorenylmethyl succinimidyl carbonate (Fmoc-OSu) was purchased from Iris Biotech GmbH (Marktredwitz, Germany). Ethyl acetoacetate as a starting substrate in Method 2 was from Sigma-Aldrich (St. Louis, MO, USA). The zinc dust and *N,N*-diisopropylethylamine were purchased from Fluka (Steinheim, Germany). Sodium nitrite was obtained from POCh and glacial acetic acid from J. T. Baker (Deventer, The Netherlands). The inorganic salts such as potassium hydrogen sulfate and anhydrous magnesium sulfate were purchased from Chempur. The solvents for peptide synthesis (analytical grade) were obtained from Riedel de Haën (Seelze, Germany) (DMF) and J. T. Baker (methanol).

### Synthesis of Fmoc-amino(2-metyhyl-1,3-dioxolan-2-yl)acetic acid (Method 1A) and Fmoc-amino(2,5,5-trimetyhyl-1,3-dioxolan-2-yl)acetic acid (Method 1B)

#### Method 1A

##### Synthesis of Z-Thr(O)-OBzl (1)

*Oxidation 1* Z-Thr-OBzl (0.5 g, 1.5 mmol) was dissolved in dichloromethane (40 ml). After the addition of PCC (0.69 g, 0.32 mmol) and molecular sieves (3 g), the resulting mixture was stirred for 24 h. After this time, the mixture was diluted with diethyl ether (100 ml) and filtered through a short column containing silica gel and evaporated to dryness.

HPLC: retention time (min): 35.7 (conditions for HPLC are given in the “Experimental” section). HR-MS *m*/*z*: found 364.128 calculated for (C_19_H_19_NO_5_ + Na)^+^ 364.116.

*Oxidation 2* Z-Thr-OBzl (0.5 g, 1.5 mmol) was dissolved in dichloromethane (5 ml) and then triethylamine (0.6 ml. 4.3 mmol) was added. The mixture was cooled to −10 °C in the ice bath. The next step included addition of the SO_3_/pyridine complex in freshly distilled DMSO (5 ml). The resulting mixture was stirred for 2.5 h. The progress of the reaction was controlled using TLC (eluent: E2). After the reaction completion, water (20 ml) with potassium hydrogen sulfate was added to increase the salting-out effect. Then, the mixture was extracted with ethyl acetate (15 ml) and the organic layer was washed with water and brine. The combined organic extracts were dried over anhydrous MgSO_4_ and evaporated to dryness.

HPLC: retention time (min): 35.6 (conditions for HPLC are given in the “Experimental” section). HR-MS *m*/*z*: found 364.127 calculated for (C_19_H_19_NO_5_ + Na)^+^ 364.116.

##### Synthesis of Z-Thr(Amda)-OBzl (2)

The crude, oxidized product (0.51 g, 1.5 mmol) was dissolved in benzene (60 ml). To this mixture, ethylene glycol (0.36 g, 5.8 mmol) and *p*-toluenesulfonic acid (120 mg) (as a catalyst) were added (Green and Wuts 1999). The resulting mixture was refluxed for 2.5 h using a Dean-Stark apparatus to remove water. After the reaction completion, the solvent was evaporated under reduced pressure. HR-MS *m*/*z*: found 408.141 calculated for (C_21_H_23_NO_6_ + Na)^+^ 408.142.

##### Synthesis of Fmoc-Thr(Amda)-OH (3)

To remove protecting groups, the obtained crude product was subjected to hydrogenolysis in methanol, using 5 % Pd on charcoal as a catalyst. The reduction was carried out for 4 h and the reaction progress was controlled with TLC (eluent: E2). Then, the mixture was filtered through a paper filter and evaporated to dryness.

TLC: retention time *R*_f_: 0.19 (conditions for TLC are given in the “Experimental” section). HR-MS *m*/*z*: found 162.083 calculated for (C_6_H_11_NO_4_ + H)^+^ 162.076.

The last step included introduction of the fluorenylmethoxycarbonyl group (Fmoc). For this purpose, the crude product (1.5 mmol) was dissolved in water (15 ml) and brought to pH 8 with NaHCO_3_. After determination of the pH, Fmoc-OSu (0.49 g, 1.5 mmol) was added. Due to the precipitation, the stoichiometric amount of DIEA (0.25 ml) in acetone (15 ml) was added. The reaction progress was controlled using TLC (eluent: E1). After the reaction completion, acetone was removed using a rotary evaporator. The remaining solution was acidified using potassium hydrogen sulfate and then extracted with ethyl acetate (30 ml), and washed with water. Finally, the combined organic layers were dried over anhydrous magnesium sulfate and evaporated to dryness.

*Fmoc-Amda-OH* yield: 25 %; HPLC: retention time (min): 33.3 (conditions for HPLC are given in the “Experimental” section). HR-MS *m*/*z*: found 406.126 calculated for (C_21_H_21_NO_6_ + Na)^+^ 406.126; MS/MS (parent 406.126): 362.148.

*Fmoc-βAla-OH* HPLC: retention time (min): 32.5 (conditions for HPLC are given in the “Experimental” section). HR-MS *m*/*z*: found 334.105 calculated for (C_17_H_18_NO_4_ + Na)^+^ 334.105; ^1^H NMR (CDCl_3_) δ (ppm) = 7.74 (d, J = 7.5 Hz, 2H), 7.56 (d, J = 7.4 Hz, 2H), 7.38 (t, J = 7.4 Hz, 2H), 7.29 (t, J = 7.3 Hz, 2H), 5.25 (s, 1H), 4.42 (m, 2H), 3.42 (m, 2H), 2.50 (m, 2H); ^13^C NMR (CDCl_3_) δ (ppm) = 176.93, 156.58, 143.96, 141.55, 127.93, 127.27, 125.29, 120.22, 67.03, 47.43, 36.55, 34.30.

#### Method 1B

##### Synthesis of Z-Thr(Atda)-OBzl (2)

This procedure included the same synthetic step as described in Method 1A. As a protecting group, 2,2,-dimetylopropan-1,3-diol was used. The crude, oxidized product (0.51 g, 1.5 mmol) was dissolved in toluene (60 ml) and then, 2,2,-dimetylopropan-1,3-diol (0.60 g, 5.8 mmol) was added to this mixture with *p*-toluenesulfonic acid (120 mg) as a catalyst. The resulting mixture was refluxed for 2.5 h using a Dean-Stark apparatus to remove water. After the reaction completion, the solvent was evaporated under reduced pressure. HR-MS *m*/*z*: found 450.193 calculated for (C_24_H_29_NO_6_ + Na)^+^ 450.188.

##### Synthesis of Fmoc-Thr(Atda)-OH (3)

The last two steps consisted of hydrogenolysis and introduction of the Fmoc group as described in Method 1A. Finally, the reaction product was purified by chromatography on a silica gel. Impurities were eluted first using chloroform containing 3 % of methanol, then, the reaction product was eluted using chloroform containing 3 % of methanol and 0.15 % of acetic acid.

Yield: 60 %; HPLC: retention time (min): 31.5 (conditions for HPLC are given in the “Experimental” section). HR-MS *m*/*z*: found 448.172 calculated for (C_24_H_27_NO_6_ + Na)^+^ 448.173; MS/MS (parent 448.171): 404.182; ^1^H NMR (CDCl_3_) δ (ppm) = 7.74 (d, J = 7.5 Hz, 2H), 7.58 (t, J = 7.2 Hz, 2H), 7.37 (t, J = 7.4 Hz, 2H), 7.29 (td, J = 7.4 Hz, 0.9 Hz, 2H), 5.62 (d, J = 9.1 Hz, 1H), 4.63 (d, J = 9.3 Hz, 1H), 4.39 (p, J = 10.6 Hz, 2H), 4.22 (t, J = 7.1 Hz, 1H), 3.67 (dd, J = 45.9 Hz, 11.5 Hz, 2H), 3.55 (dd, J = 45.9 Hz, 11.5 Hz, 2H), 1.51 (s, 3H), 1.10 (s, 3H), 0.82 (s,3H); ^13^C NMR (CDCl_3_) δ (ppm) = 171.01, 156.55, 143.97, 141.50, 127.32, 127.29, 125.38, 120.17, 70.26, 70.23, 67.65, 59.87, 47.31, 30.24, 23.13, 22.56, 16.57.

### Synthesis of Fmoc-amino(2,5,5-trimetyhyl-1,3-dioxolan-2-yl)acetic acid (Method 2)

#### Synthesis of Ac-Thr(O)-OEt (1*)

Ethyl acetoacetate (1 g, 7.63 mmol) was placed in two-necked, round bottom flask equipped with a thermometer and a magnetic stirrer. After cooling in the ice bath, glacial acetic acid (1.4 ml) and water (2 ml) were added with stirring. Sodium nitrite (1.58 g, 45.8 mmol) was added in portions over 1.5 h with the temperature kept constant at about 5 °C. Then, the ice bath was removed and the stirring was continued for 4 h. At that time, the temperature increased to 34–38 °C within 2 h and then decreased to about 29 °C. The reaction product was extracted with diethyl ether (3 × 10 ml). The combined organic layers were used directly in the next step. The obtained ether solution containing acetic anhydride (2.1 g, 20.6 mmol) and glacial acetic acid (5.78 g, 96.2 mmol) was placed in two-necked, round bottom flask equipped with thermometer. Then, the zinc dust (1.92 g, 293 mmol) was added in small portions over a period of 1.5 h with vigorous stirring at temperature in the range of 40–50 °C. The exothermic reaction was cooled in a water bath. After completion of the metal addition, the mixture was stirred for an additional 30 min. The excess zinc powder was filtered and washed thoroughly with three 10 ml portions of glacial acetic acid. The resulting filtrate was evaporated under reduced pressure and finally, thick oil was obtained.

HPLC: retention time (min): 8.8 (conditions for HPLC are given in the “Experimental” section). HR-MS *m*/*z*: found 210.081 calculated for (C_8_H_13_NO_4_ + Na)^+^ 210.074; ^1^H NMR (CDCl_3_) δ (ppm) = 1.29 (t, J = 7.1 Hz, 3H), 2.04 (s, 3H), 2.36 (s, 3H), 4.25 (qd, J = 6.0 Hz, 3.0 Hz, 2H), 5.22 (d, J = 6.5, 1H); ^13^C NMR (CDCl_3_) δ (ppm) = 14.19, 28.33, 62.88, 63.37, 166.30, 170.09, 198.87.

#### Synthesis of Ac-Thr(Atda)-OEt (2*)

The carbonylated product (0.5 g, 2.6 mmol) was dissolved in toluene (60 ml) and 2,2-dimethyl-propane-1,3-diol (1.08 g, 10.3 mmol), and *p*-toluenesulfonic acid as a catalyst (150 mg) were added. The resulting mixture was refluxed for 2.5 h using a Dean-Stark apparatus. After the reaction completion, the solvent was evaporated under reduced pressure (Green and Wuts 1999). HR-MS *m*/*z*: found 296.141 calculated for (C_13_H_23_NO_5_ + Na)^+^ 296.147.

#### Synthesis of Fmoc-Thr(Atda)-OH (3*)

To remove the C and N protecting groups, the alkaline hydrolysis with 3.5 M NaOH was carried out for 48 h. The reaction was controlled with a ninhydrin test. After the hydrolysis was completed, the resulting strongly alkaline mixture was used in the next step. It was brought to pH 8 with concentrated hydrochloric acid and treated with Fmoc-OSu (0.90 g, 2.56 mmol) in acetone, to reach the conditions for introducing the Fmoc-protecting group described in this paper (Method 1A or 1B). Finally, the reaction product was purified as described in Method 1B.

Yield: 60 %; HPLC: retention time (min): 31.5 (conditions for HPLC are given in the Experimental section). HR-MS *m*/*z*: found 448.173 calculated for (C_24_H_27_NO_6_ + Na)^+^ 448.173; MS/MS (parent 448.173) 404.180.

### Peptide preparation

The model peptides were prepared on a solid support according to the standard Fmoc protocol (Chan and White 2000). The coupling of respective amino acid residues was carried out using TCTU in DMF. The acetal protection of the carbonyl group was removed simultaneously with the peptide cleavage from the resin, using TFA/H_2_O/TIS (95:2.5:2.5, v/v) for 3 h at room temperature, resulting in a carbonylated peptide.

### Purification and characterization of peptides

The crude peptide products after releasing from the resin were analyzed using a Thermo Separation HPLC system with a UV detection (210 nm) on a Vydac Protein RP C18 column (4.6 × 250 mm, 5 μm), with a gradient elution of 0–80 % S2 in S1 (S1 = 0.1 % aqueous TFA in H_2_O; S2 = 80 % acetonitrile + 0.1 % TFA) for 40 min (flow rate 1 ml/min). The main carbonylated product was purified using preparative reversed-phase HPLC on a TOSOH Bioscience TSKgel ODS 120T column (21.5 mm × 300 mm; 10 μm), using eluent systems: S1 0.1 % aqueous TFA, S2 80 % acetonitrile + 0.1 % TFA, linear gradient from 50 to 100 % of S2 for 40 min, flow rate 7.0 ml/min, UV detection at 220 nm. The resulting fractions were collected and lyophilized. The identities of the products were confirmed by MS analysis using a microTOF-Q mass spectrometer equipped with an electrospray ionization source.

### TLC analysis

The reactions described in this paper were controlled using TLC. This procedure was performed using TLC plates (Pre-coated TLC plates ALUGRAM^®^ SIL G/UV_254_, 5 × 10 cm) and eluent systems: *n*-buthyl alcohol:acetic acid:water (4:1:1) as an eluent E1; chloroform:methanol (95:5) as an eluent E2. The chromatograms were visualized using a UV lamp (LAMAG) at 254 nm.

### Mass spectrometry measurements

Mass spectrometric measurements were performed on a quadrupole time-of-flight (micrOTOF-Q) instrument (Bruker, Germany) equipped with an electrospray ionization source. The instrument was operated in the positive or negative ion mode and calibrated before each analysis with the Tunemix mixture (Bruker Daltonics) by a quadratic method. In the MS/MS experiments, the collision energy (5–20 eV) was optimized for the best fragmentation. The solvent used for recording the mass spectra was acetonitrile:water:formic acid (50:50:0.1) mixture or methanol. The potential between the spray needle and the orifice was set to 4.5 kV. In the MS/MS mode, the quadrupole was used to select the precursor ions, which were fragmented in the hexapole collision cell generating product ions that were subsequently mass analyzed by the orthogonal reflectron TOF mass analyzer. For the collision-induced dissociation (CID) MS/MS measurements, the voltage over the hexapole collision cell varied from 15 to 30 V and argon was used as a collision gas.

### LC–MS

The LC–MS analysis was performed on an Agilent 1200 HPLC system coupled to a micrOTOF-Q system mass spectrometer (Bruker Daltonics, Germany). Separation was carried out on an RP-Zorbax (50 × 2.1 mm, 3.5 µm) column with a gradient elution of 0–80 % B in A (A, 0.1 % HCOOH in water; B, 0.1 % HCOOH in acetonitrile) at room temperature (flow rate: 0.1 ml/min) over 40 min.

### Circular dichroizm

The CD spectrum was recorded on a Jasco J-600 spectropolarimeter. Fmoc-Atda-OH was dissolved in methanol at a concentration of 0.07 mg/ml. The spectrum of the solvent was recorded under identical conditions and subtracted during data analysis. A rectangular quartz cell of 1 mm pathlength was used. The data are presented as a mean residue molar ellipticity [ϴ]. The sample was measured in the range from 260 to 195 nm.

## Results and discussion

### Synthesis of Fmoc-Amda-OH (Fmoc-amino(2,5,5-trimethyl-1,3-dioxolan-2-yl)acetic acid)

**Fig. 1:**
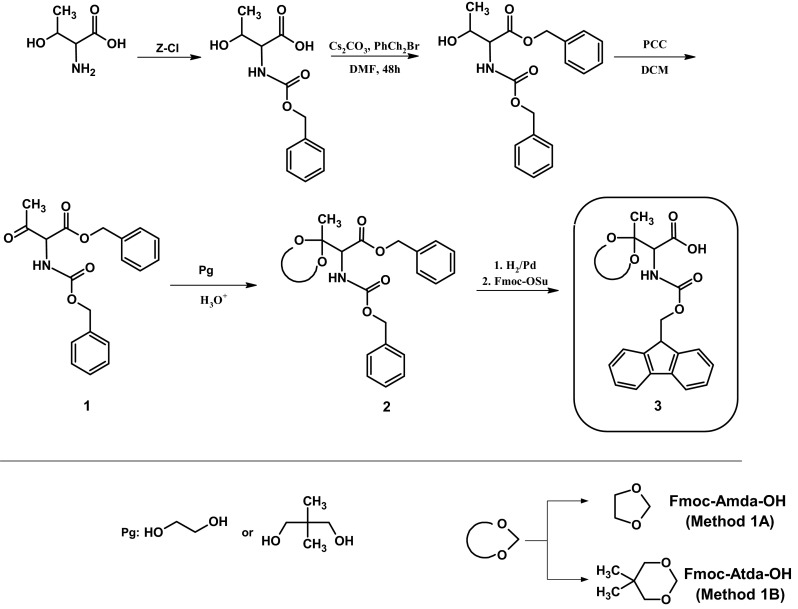
Synthesis of Fmoc-Amda-OH (Method 1A) and Fmoc-Atda-OH (Method 1B)

Herein we propose a new method for synthesis of an unnatural fully protected amino acid for the solid phase synthesis of carbonylated peptides according to the standard Fmoc protocol. So far, only one product, protected aminoadipic semialdehyde, which is a derivative of the oxidized lysine moiety, is commercially available. However, to the best of our knowledge, there are no reports on successful application of this derivative in the peptide synthesis. We performed the synthesis of a modified amino acid (fully protected, oxidized threonine), according to the scheme presented in Fig. 1a. After protecting the *N*-amino and carboxylic groups, the side chain of threonine was oxidized using pyridinium chlorochromate (PCC) (Corey and Suggs 1975) for 24 h at room temperature. The alternative reagent that allows obtaining the same carbonylated compound was the SO_3_/pyridine complex in DMSO. Our attempts at a direct synthesis of Fmoc-Amda-OH by oxidation of Fmoc-Thr-OH by PCC failed because of fast decarboxylation of the reaction product. Therefore, we examined the protection of the carbonyl group using ethylene glycol. After deprotection of the α-amino and carboxylic groups by catalytic hydrogenation in methanol, the Fmoc group was introduced. Our results show that synthesis of the oxidized derivative of threonine carried by the proposed method is feasible. In the ESI–MS spectrum of crude Fmoc-Amda-OH, a signal derived from the desired product is observed (Fig. S1A Supplementary Materials). The spectrum is dominated by two peaks. Next to the peak at *m*/*z* 406.126, corresponding to the sodium adduct of a carbonylated amino acid, there is another peak at *m*/*z* 334.105. Our further studies revealed that this signal corresponds to Fmoc-βAla-OH. The yield of the desired product is relatively low while its retention time is close to that of the by-product (Fig. S1B Supplementary Materials).

**Fig. 2:**
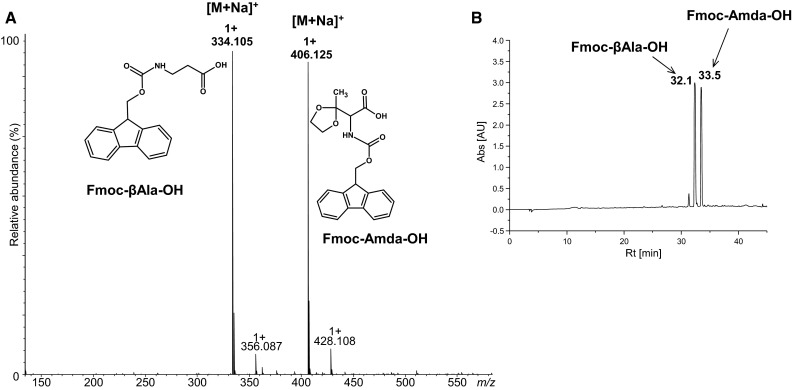
ESI–MS spectrum (**a**) and chromatogram (**b**) of the purified product (Synthesis of Fmoc-Amda-OH Method 1A)

The reaction product was purified by liquid chromatography on a silica gel, using 5 % isopropanol in chloroform as an eluent. Unfortunately, separation of Fmoc-Amda-OH and Fmoc-βAla-OH on a preparative scale was not efficient because of coelution of these compounds (Fig. 2b). The ESI–MS spectrum (Fig. 2a) of the crude product is dominated by two peaks corresponding to Fmoc-βAla-OH and Fmoc-Amda-OH. The presented chromatograms (Fig. 2b) confirmed only the purity of the by-product.

Our investigations confirmed that the by-product obtained is Fmoc-βAla-OH. In the ESI–MS spectrum (Fig. S2A Supplementary Materials), only one peak corresponding to this product is observed. The structure of this compound was confirmed by NMR (Fig. S2B, S3, S4 Supplementary Materials). Obkircher et al. (2008) suggested previously that β-alanine may be formed by the Lossen rearrangement during the introduction of the Fmoc-protecting group.

As the yield of the synthesis was low and the separation of Fmoc-Amda-OH and Fmoc-βAla-OH on a preparative scale was not feasible, we decided to change the carbonyl group protection, therefore, a second synthetic strategy was proposed (Fig. 1b). This procedure included the same synthetic steps, but that time the carbonyl group was protected by 2,2-dimethylpropane-1,3-diol. According to the literature, this protecting group should be more labile at acidic conditions (Newmann and Harper 1958). We also expected that the new, more hydrophobic protection will facilitate chromatographic purification of the reaction product.

**Fig. 3:**
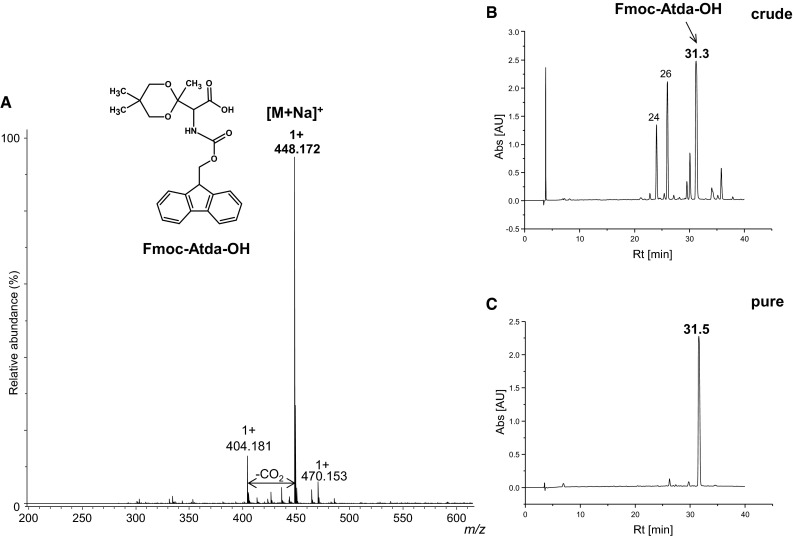
ESI–MS (**a**) of pure Fmoc-Atda-OH; chromatogram of crude (**b**) and pure (**c**) product obtained by Method 1B

The result of the synthesis is presented in Fig. 3. In the ESI–MS spectrum, the signal of the desired product is observed. The product was successfully purified and only one peak corresponding to fully protected oxidized threonine is present in the chromatogram of the purified fraction. The structure of this building block was confirmed by NMR (Fig. S5, S6 Supplementary Materials). During the synthesis of Fmoc-Atda-OH using 2,2-dimethylpropane-1,3-diol as a protecting group, the amount of obtained Fmoc-β-Ala-OH was negligible. These data may be explained basing on the literature (Obkircher et al. 2008). The authors investigated the amount of Fmoc-βAla-OH formed from Fmoc-OSu, in relation to the amino acid residue being protected. It seems that the branched amino acids influence the formation of Fmoc-βAla-OH by-product during Fmoc protection reaction. The content of impurity depended on the amino acid structure and reaction conditions. The by-product was formed as result of Lossen rearrangement of succinimidyl moiety; however, the influence of amino acid structure on the Fmoc- β-Ala-OH content in the crude product was not completely resolved.

**Fig. 4:**
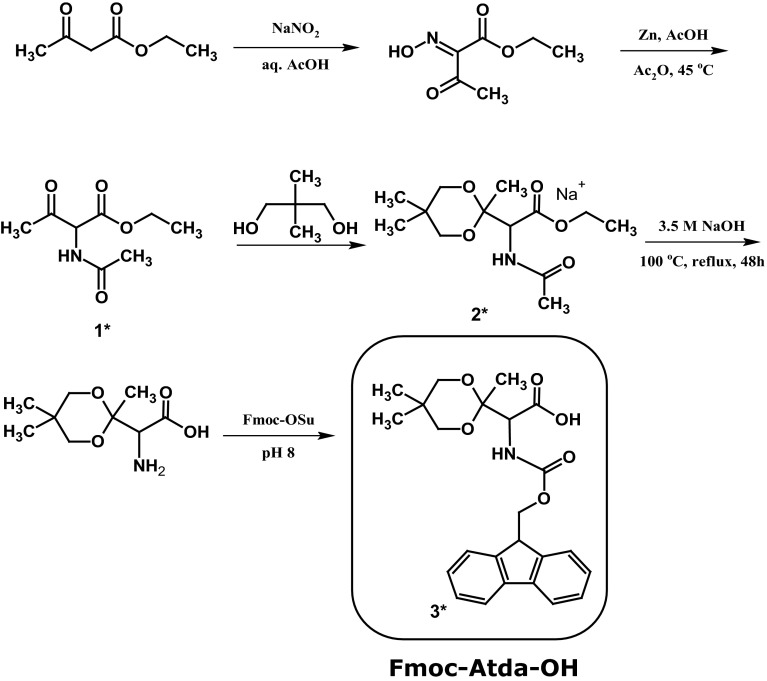
Synthesis of Fmoc-Atda-OH (Method 2)

**Fig. 5:**
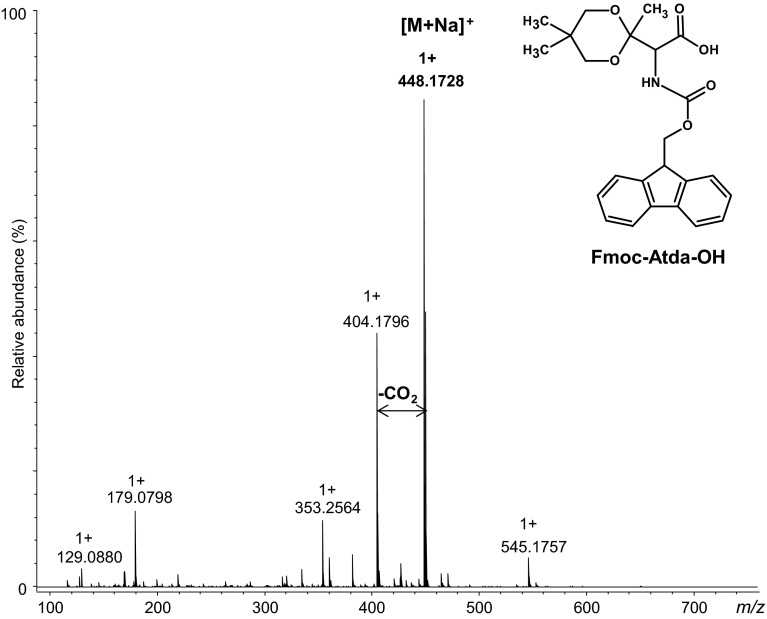
ESI–MS of crude product Fmoc-Atda-OH obtained by Method 2

In addition, we designed another synthetic strategy utilizing ethyl acetoacetate. After nitrosylation of ethyl acetoacetate, the reduction in the presence of zinc dust and acetic acid combined with a direct acetylation was performed. It allows obtaining the ethyl ester of *N*-acetylated 2-amino-3-ketobutyric acid with a high yield and purity. The carbonyl group in the obtained product was protected using 2,2-dimethyl-propan-1,3-diol. Then, alkaline hydrolysis (3.5 M NaOH for 48 h) was performed. Finally, the *N*-amino group was protected by the Fmoc group. This synthetic pathway is presented in Fig. 4. Purity and identity of the product were confirmed by HPLC and HR-MS (Fig. 5). All the *m*/*z* values were consistent with the calculated ones based on chemical formulas of the expected compounds. Based on the obtained analytical data, we proved that the desired product was obtained.

### Chirality

**Fig. 6:**
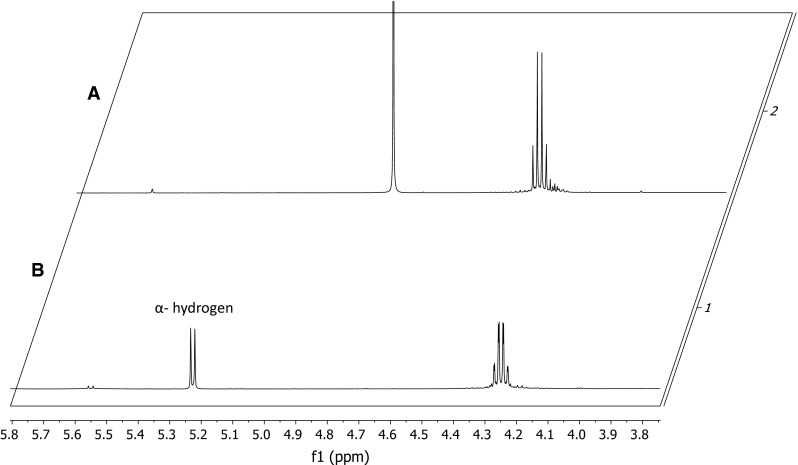
^1^H NMR spectra of Ac-Thr(O)-OEt measured in: *a* D_2_O after 3 min; *b* CDCl_3_

^1^H NMR experiments using Ac-Thr(O)-OEt were carried out in D_2_O and CDCl_3_, to check the dependence of the exchange rate of α-hydrogen on solvent and time. The study revealed that the hydrogen–deuterium exchange was relatively fast. The peak corresponding to α-hydrogen was not present in the ^1^H NMR spectra obtained 3 min after dissolving the sample in D_2_O, while the sample dissolved in de-acidified chloroform showed a doublet corresponding to α-hydrogen (Fig. 6, S7 Supplementary Materials). This result reveals a lability of the α-carbon proton which may be explained by formation of enolic form characteristic for dicarbonyl systems. Fmoc-Atda-OH was subjected to CD study. The CD spectrum presented in Fig. S8 (Supplementary Materials) did not show any ellipticity in the range from 260 to 190 nm. Observed racemization is likely a result of planar enol formation.

### Synthesis of carbonylated peptides

Pure, fully protected derivative of oxidized threonine (Fmoc-D,L-Atda-OH) was applied in the solid phase synthesis of carbonylated peptides. Peptides were prepared by manual solid phase technique using the standard Fmoc synthetic strategy with TBTU as a coupling reagent. The peptide was cleaved from the resin using TFA/water/TIS (95:2.5:2.5, v/v).

**Fig. 7:**
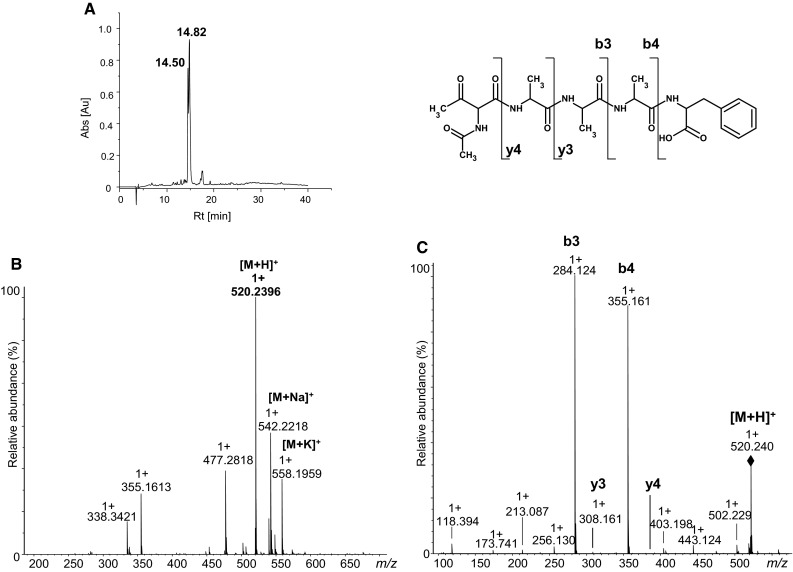
Analytical data of Ac-Thr(O)-Ala-Ala-Ala-Phe-OH (Method 2). **a** Chromatogram; **b** ESI–MS spectrum; **c** ESI–MS/MS spectrum

A series of synthetic model peptides containing the building block (H-Thr(O)-Ala-Phe-OH, H-Thr(O)-Ala-Ala-Ala-Phe-OH, Ac-Thr(O)-Ala-Ala-Ala-Phe-OH, H-Gly-Thr(O)-Ala-Ala-Ala-Phe-OH, H-Leu-Val-Asn-Glu-Val-Thr(O)-Glu-Phe-Ala-Lys-OH) were synthesized. To make this model more realistic, we based the sequence of one peptide on a fragment of naturally occurring protein (tryptic fragment [66–75] HSA containing Thr residue). The oxidized threonine residue replaces a threonine residue in a natural protein giving the sequence: H-Leu-Val-Asn-Glu-Val-Thr(O)-Glu-Phe-Ala-Lys-OH. The influence of position of the unnatural amino acid in the peptide chain on efficiency of deprotection of the carbonyl group was studied. In the case of application of 2,2-dimethylpropane-1,3-diol as a protecting group, we found that incorporation of a carbonylated amino acid at the N-terminus does not allow removal of the masking group even after 24 h incubation. However, the elongation of carbonylated peptide (H-Gly-Thr(O)-Ala-Ala-Ala-Phe-OH) or acetylation of the N-terminus (Ac-Thr(O)-Ala-Ala-Ala-Phe-OH) allowed a complete removal of the protecting group from carbonyl in 3 h (Fig. 7a). Application of this derivative resulted in obtaining of the carbonylated peptides with a 90 % yield. A signal of the protonated, carbonylated peptide is observed in the MS spectrum of the crude product (Fig. 7b). The parent ion at *m*/*z* 520.239, was subjected to MS/MS fragmentation. The fragmentation spectrum is dominated by a series of b and y ions covering the whole sequence of the peptide (Fig. 7c, S9 Supplementary Materials). In the chromatogram of a pure carbonylated peptide, two signals corresponding to diastereomers were observed because of racemization.

We also performed a successful synthesis of a tryptic fragment [66–75] HSA containing carbonylated threonine (H-Leu-Val-Asn-Glu-Val-Thr(O)-Glu-Phe-Ala-Lys-OH). The ESI–MS and ESI–MS/MS spectra, and the HPLC chromatogram of pure H-Leu-Val-Asn-Glu-Val-Thr(O)-Glu-Phe-Ala-Lys-OH are presented (Fig. S10 Supplementary Materials).

In the synthesis, we applied a racemic derivative Fmoc-D,L-Atda-OH, obtaining a mixture of diastereoisomeric peptides. However, the composition of this product is a matter of equilibrium and does not depend on the chirality of applied substrate (Fmoc-Atda-OH).

**Fig. 8:**
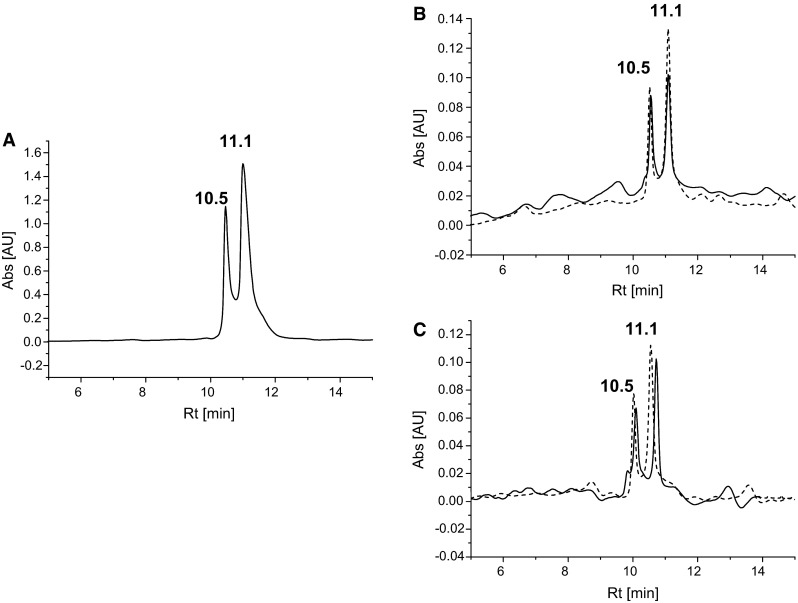
**a** Chromatogram of pure H-Leu-Val-Asn-Glu-Val-Thr(O)-Glu-Phe-Ala-Lys-OH; **b** chromatograms of fractions corresponding to each peak reanalyzed 30 min after separation (*solid line* the fraction eluted at 10.5 min; *dotted line* the fraction eluted at 11.1 min); **c** chromatograms of fractions corresponding to each peak reanalyzed 24 h after separation (*solid line* the fraction eluted at 10.5 min; *dotted line* the fraction eluted at 11.1 min

We tested a progress of racemization as a function of time for peptide H-Leu-Val-Asn-Glu-Val-Thr(O)-Glu-Phe-Ala-Lys-OH using HPLC. Two signals corresponding to two isomeric forms of the pure peptide are observed in the chromatogram (Fig. 8a). The fractions corresponding to both peaks were collected and subjected to re-analysis of purity at the same conditions. The obtained chromatograms showed that two isomeric forms appeared after less than 30 min (Fig. 8b). The HPLC analysis executed after 24 h for the separated signals showed the same peak areas (Fig. 8c).

**Fig. 9:**
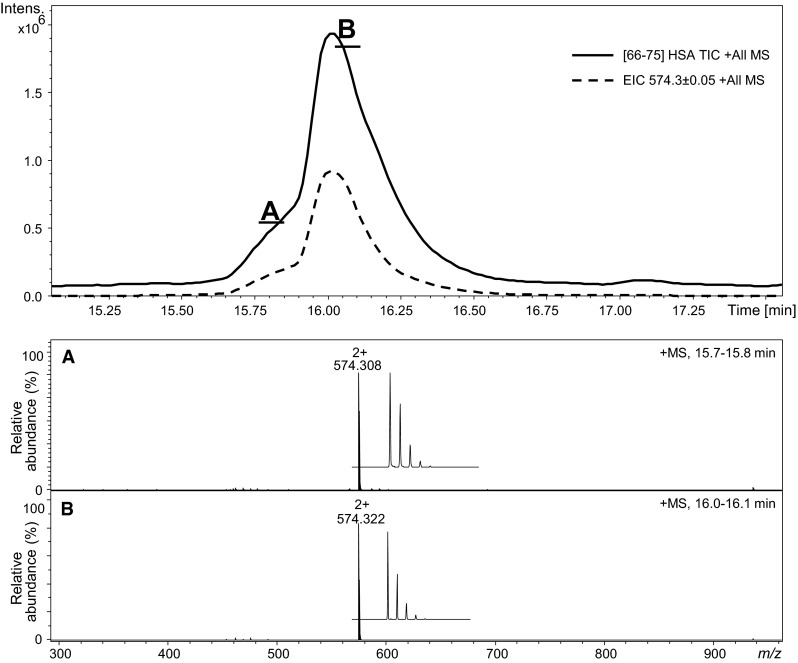
LC–MS of pure HSA [66–75]; **a** ESI–MS of the peptide eluted at 15.7–15.8 min; **b** ESI–MS of the peptide eluted at 16.0–16.1 min

LC–MS analysis confirmed that two peaks in the chromatogram correspond to compounds with the same molecular weight (Fig. 9). The resolution of the column used for LC was not sufficient to completely separate these two signals. However, the presented spectra (Fig. 9a, b) revealed unquestionably the same *m*/*z* for the appropriate parts of peaks. Additionally, the extracted ion chromatogram was generated by Data Analysis program and exactly the same profile of the chromatogram was obtained. In the chromatogram (Figs. 8, 9), the peaks corresponding to two diastereoisomeric forms of modified peptides are characterized by broadening and tailing. These features additionally confirmed our results concerning the fast equilibrium between both stereoisomeric peptides. Based on the NMR study, we confirmed the exchange rate of α-hydrogen. Theoretically, the effect may result from the presence of an enol form. Additionally, the *cis*-/*trans*-isomerizatiom on the peptide bond may contribute to a further peak boarding; however, a thorough investigation is needed to confirm this speculation.

We also applied a crude, fully protected amino acid containing ethylene glycol as a protection of the carbonyl group (Fmoc-Amda-OH) for the solid phase synthesis of carbonylated peptides. Peptides were prepared as previously described. Our results indicate that Fmoc-Amda-OH was also successfully incorporated into model peptides. The ESI–MS spectrum of crude product Ac-Thr(O)-Ala-Ala-Ala-Phe-OH is presented (Fig. S11 Supplementary Materials). In the spectrum, three main peaks correspond to the protonated peptides containing β-alanine, Thr(O), and Amda [protected Thr(O)]. After extending the time of cleavage of the peptide, the deprotection was complete (see below) and the signal at *m*/*z* 564.267 was not observed. We found that incorporation of a carbonylated amino acid at the N-terminus does not allow removing the acetal group even after 24 h of incubation, in the mixture used for cleavage or microwave-assisted TFA (Kluczyk et al. 2010) cleavage from the resin. The probable reason is the proximity of the amino group which easily gets protonated. The positive charge on the nitrogen prevents protonation of acetal oxygen because of possible electrostatic repulsion. The elongation of peptide at the N-terminus allowed the removal of the acetal group after 8 h of incubation. Acetylation of the N-terminal amino group decreased the time of complete cleavage to 4 h. The purity of synthesized peptides was checked by HPLC. The chromatograms of the crude product after various times of cleavage are presented (Fig. S12A Supplementary Materials). As one can see, after 4 h of incubation, the acetal group is completely removed from the carbonyl group. Two main products of synthesis were successfully separated using HPLC. The chromatograms of pure compounds are presented (Fig. S12B and C Supplementary Materials).

We observed the racemization also in the case of Fmoc-Amda-OH synthesis. The racemization is a result of chemical structure of oxidized threonine derivative. The proton at α carbon is activated by two carbonyl groups and undergoes fast exchange. The chromatograms of crude and pure carbonylated peptides (Ac-Thr(O)-Ala-Ala-Ala-Phe-OH) prepared by solid phase synthesis using crude Fmoc-Amda-OH were placed in Supplementary Materials (Fig. S12A and C Supplementary Materials). Two fractions corresponding to these signals were collected and analyzed by mass spectrometry, showing the same molecular mass, which is in good agreement with assumption that Thr(O) residue is susceptible to racemization.

## Conclusions

We designed a straightforward and convenient method of synthesis of the fully protected carbonylated building block Fmoc-Atda-OH. Two different protections of the carbonyl group were tested. The application of 2,2-dimethylpropane-1,3-diol results in a better yield and purity of the fully protected building block as compared to ethylene glycol. The crude Fmoc-Amda-OH and pure Fmoc-Atda-OH were successfully applied in the solid phase synthesis of carbonylated peptides. The kinetics of the carbonyl group deprotection with respect to the position of carbonylated amino acid residue in the peptide chain was investigated, revealing that peptides with N-terminal Thr(O) are not susceptible to deprotection with trifluoroacetic acid. In addition, the main by-product (Fmoc-βAla-OH) formed during Fmoc-Amda-OH was identified.

## Electronic supplementary material

Below is the link to the electronic supplementary material.

Supplementary material 1 (DOCX 789 kb)
