# Supplementary material for: Site-selective solid phase synthesis of carbonylated peptides
Source: Amino Acids. 2015 Mar 27;47(7):1353–65. doi: 10.1007/s00726-015-1967-4 (PMC4458268; doi:10.1007/s00726-015-1967-4)
Supplement: Supplementary file 1 — Supplementary material 1 (DOCX 789 kb) [file 726_2015_1967_MOESM1_ESM.docx]

**Supplementary Materials Amino Acid**

**Site-selective solid phase synthesis of carbonylated peptides**

Mateusz Waliczek, Monika Kijewska, Piotr Stefanowicz, Zbigniew Szewczuk

*Faculty of Chemistry, University of Wrocław*

**Corresponding author:** Monika Kijewska, Faculty of Chemistry, University of Wrocław, F. Joliot-Curie 14, 50-383 Wrocław, Poland, Fax: +48‑71‑3282348, Tel.: +48-71-3757213, E‑mail: monika.kijewska@chem.uni.wroc.pl

**Fig. S1** ESI-MS spectrum (A) and chromatogram (B) of crude product (Synthesis of Fmoc-Amda-OH Method 1A) (circle – retention time of Fmoc-Amda-OH, square – retention time of Fmoc-βAla-OH)

**Fig. S2** Analytical data for Fmoc-βAla-OH (A – 2D ^1^H-^1^H NMR spectrum; B – ESI-MS spectrum; C – Chromatogram)

**Fig. S3** ^1^H NMR (CDCl_3_) spectrum of Fmoc-βAla-OH

**Fig. S4** ^13^C NMR (CDCl_3_) spectrum of Fmoc-βAla-OH

**Fig. S5** 1H NMR (CDCl_3_) spectrum of Fmoc-Atda-OH

**Fig. S6** ^13^C NMR (CDCl_3_) spectrum of Fmoc-Atda-OH

**Fig. S7** ^1^H NMR spectra for Ac-Thr(O)-OEt measured in: A - D_2_O after 3min; B - D_2_O after few days; C - CDCl_3_

**Fig. S8** The CD spectrum of Fmoc-Atda-OH measured in methanol

**Fig. S9** ESI-MA/MS of Ac-Thr(O)-Ala-Ala-Ala-Phe-OH (expanded range)

**Fig. S10** ESI-MS (A) and ESI-MS/MS (B) spectra for the pure H-Leu-Val-Asn-Glu-Val-Thr(O)-Glu-Phe-Ala-Lys-OH

**Fig. S11** ESI-MS of crude Ac-Thr(O)-Ala-Ala-Ala-Phe-OH (time of cleavage 2h)

**Fig. S12** A) Chromatogram of crude Ac-Thr(O)-Ala-Ala-Ala-Phe-OH after various time of cleavage; B) chromatogram of pure Ac-βAla-Ala-Ala-Ala-Phe-OH; C) chromatogram of pure Ac-Thr(O)-Ala-Ala-Ala-Phe-OH
